# Spontaneous bone infarction of the distal femur in a patient with Cushing's disease: a case report

**DOI:** 10.1016/j.bonr.2021.100756

**Published:** 2021-02-18

**Authors:** Pepijn van Houten, Jacky de Rooy, Ingrid van der Geest, Romana Netea-Maier, Annenienke van de Ven

**Affiliations:** a Department of Internal Medicine, Division of Endocrinology, Radboud University Medical Centre, Nijmegen, the Netherlands; b Department of Radiology and Nuclear Medicine, Radboud University Medical Centre, Nijmegen, the Netherlands; c Department of Orthopedic Oncologic Surgery, Radboud University Medical Centre, Nijmegen, the Netherlands

**Keywords:** CS, Cushing's syndrome, AVN, avascular necrosis, DST, 1 mg dexamethasone suppression test, n, normal value, ACTH, adrenocorticotropic hormone, MRI, magnetic resonance imaging, ALL, acute lymphoblastic leukemia, LMWH, low-molecular weight heparin, Cushing's disease, Bone infarction, Avascular necrosis, Osteonecrosis

## Abstract

Avascular necrosis of the femoral head is a well-known complication of treatment with high dosage glucocorticoids and has been described in a few patients with Cushing's syndrome. In this case report, we describe the, to our knowledge, first case of a patient with endogenous Cushing's syndrome with a bone infarction located in the distal femur. In patients with Cushing's syndrome and bone pain, the diagnosis of bone infarction should be considered as it can occur as a rare complication of hypercortisolism.

## Highlights

• Avascular necrosis of the femoral head is a known complication of glucocorticoids.
• Bone infarction of the distal femur is less prevalent.
• Bone infarction can also occur in endogenous Cushing's syndrome.
• Bone infarction should be considered in cases of Cushing's syndrome and bone pain.

## 1 Introduction

The use of glucocorticoids is associated with the development of bone infarction, also known as osteonecrosis or avascular necrosis (AVN). Avascular necrosis of the femoral head, which is one of the most commonly affected localizations, is a well-known complication of treatment with high dosage glucocorticoids and has been described in a few patients with endogenous Cushing's syndrome (Belmahi et al., 2018; Bukhman et al., 1987; Camporro et al., 2016; Cerletty et al., 1973; Güven et al., 2009; Ko et al., 2004; Kobayashi and Terayama, 1991; Koch et al., 1999; Modroño et al., 2014; Pazderska et al., 2016; Phillips et al., 1986; Premkumar et al., 2013; Sharon et al., 1977; Takada et al., 2004; Wicks et al., 1987). Here, we present a case of a 22-year old male patient with severe hypercortisolism due to Cushing's disease, who developed a spontaneous bone infarction of the distal femur.

## 2 Case description

A 22-year old male patient with no previous medical history was referred to our tertiary center with suspicion of Cushing's syndrome. During the last two years, he suffered from complaints of fatigue, gain of weight (10 kg) and psychological problems. Physical examination showed a cushingoid appearance with full moon face, supraclavicular fat pads, abdominal obesity, proximal muscle wasting, reddish-purple striae at the abdomen and upper arms, cutaneous fungal infection and hematomas. Biochemical analysis confirmed ACTH dependent severe hypercortisolism: cortisol concentration after 1 mg dexamethasone suppression test (DST) was 0.61 μmol/l (normal value (n) <0.05 μmol/l), 24-h urine free cortisol level was 2260 nmol (n < 150 nmol), late night salivary cortisol concentration was 39.0 nmol/l (n < 3.0 nmol/l) and adrenocorticotropic hormone (ACTH) concentration was 28.8 pmol/l (n 1.6–13.9 pmol/l) (Nieman et al., 2008). Pituitary MRI showed a lesion of 8 × 5 mm in the anterior pituitary gland and the patient was referred to the neurosurgeon for resection of the adenoma. Endoscopic endonasal transsphenoidal surgery was performed followed by remission of Cushing's syndrome. Pathological examination of the resected tissue showed an ACTH-producing adenoma.

For 5 months prior to the diagnosis of Cushing's disease, the patient suffered from pain in his left upper leg, for which he was referred to the orthopedic surgeon. Physical examination showed normal knee function without swelling or pain. There was no axial pressure pain in the upper leg.

**Fig. 1:**
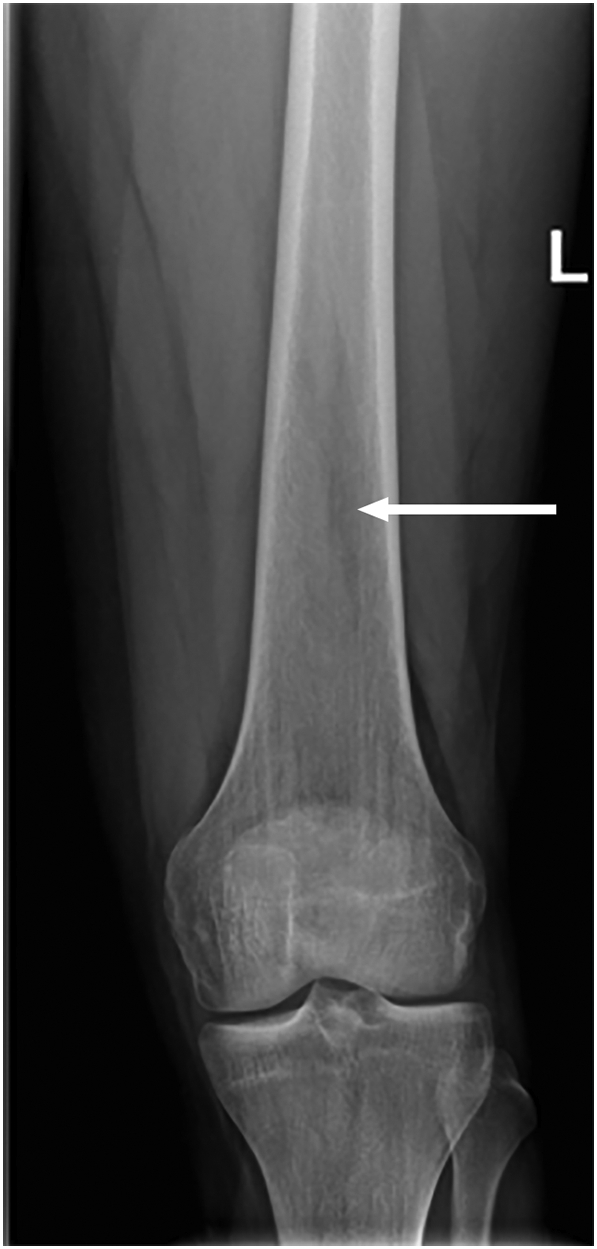
Conventional X-ray of the left distal femur, showing intramedullary sheath-like lucencies in the distal femoral diaphysis.

**Fig. 2:**
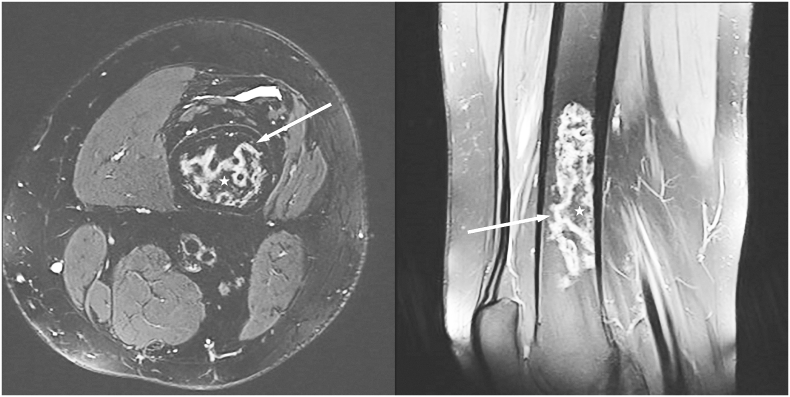
T2 weighted axial (left) and proton density weighted sagittal (right) MR images of the left distal femur with fat saturation. Serpenginous intramedullary high signal lines (arrows) surrounding normal fatty marrow (asterix) in the distal femoral diaphysis.

Conventional X-ray of the distal femur showed a probable sheath-like intramedullary lucency, which was only recognized in retrospect after magnetic resonance imaging (MRI) (Fig. 1). MRI showed a 10 cm craniocaudal area of serpiginous unsharp PD- and T2-hyperintense and T1-intermediate intense lines surrounding normal fatty bone marrow in the distal femoral metadiaphysis (Fig. 2). There were no peripheral low-signal intensity lines, confirming lack of sclerosis on conventional X-rays and consistent with early bone marrow infarction.

The bone infarction was treated conservatively. Six months later, a follow-up MRI showed a much smaller abnormal intramedullary area of sharp T2-hyperintense and T1-hypointense lines, consistent with healing bone infarction.

## 3 Discussion

To our knowledge this is the first described case of a patient with endogenous Cushing's syndrome and bone infarction located in the distal femur. However, avascular necrosis of the femoral head has been described in some patients with endogenous Cushing's syndrome (Belmahi et al., 2018; Bukhman et al., 1987; Camporro et al., 2016; Cerletty et al., 1973; Güven et al., 2009; Ko et al., 2004; Kobayashi and Terayama, 1991; Koch et al., 1999; Modroño et al., 2014; Pazderska et al., 2016; Phillips et al., 1986; Premkumar et al., 2013; Sharon et al., 1977; Takada et al., 2004; Wicks et al., 1987). Cushing's syndrome is a rare endocrine disorder that is characterized by signs and symptoms of hypercortisolism. It is most commonly caused by an ACTH producing adenoma of the anterior pituitary gland, which is known as Cushing's disease (Lindholm et al., 2001). Less common etiologies of Cushing's syndrome are cortisol producing adrenal tumors, adrenal hyperplasia and ACTH-producing ectopic tumors.

Bone infarctions are commonly reported events during treatment with high dosage glucocorticoids with a prevalence between 3% and 38%, depending on the underlying disease and the used dosage of glucocorticoids (Assouline-Dayan et al., 2002). For example, high incidences of bone infarctions are found in pediatric patients treated for acute lymphoblastic leukemia (ALL) and in pediatric patients who have undergone allogenic bone marrow transplantation (Kaste et al., 2011). Both of these groups receive high dosages of glucocorticoids. In addition, a cohort study in patients with different kind of chronic inflammatory diseases showed an association between glucocorticoid exposure and avascular necrosis, especially in young adults (Horton et al., 2017). Other risk factors include: alcohol abuse, hematological diseases such as sickle cell anemia or thalassemia, hypercholesterolemia, chronic renal failure, autoimmune diseases, pregnancy and hyperparathyroidism (Chan and Mok, 2012; Moskal et al., 1997; Poignard et al., 2012; Wang et al., 2003). The knee is the second most common affected localization of bone infarctions, although much less common than the hip (Chan and Mok, 2012; Mont et al., 2000). Other localizations of bone infarctions are the shoulder and ankle. The specific prevalence of bone infarctions of the distal femur is not known.

The pathogenesis of glucocorticoid induced bone infarctions is not fully understood. Hypotheses include fat cell hypertrophy, fat embolization, intravascular coagulation and osteocyte apoptosis (Chan and Mok, 2012). Fat cell hypertrophy might increase intramedullary pressure and reduce venous return, which are hypothesized to lead to bone infarctions as well (Motomura et al., 2005). One animal study has shown that treatment with the low-molecular-weight heparin (LMWH) enoxaparin (Clexane) has the potential to prevent steroid-induced bone infarctions (Beckmann et al., 2014). In the mentioned study, the bone infarctions were induced by systemic treatment with methylprednisolone. This might indicate that intravascular coagulation plays an important role in the pathogenesis of bone infarctions. Furthermore, because of down-regulation of vascular endothelial growth factor, glucocorticoids can induce hypoperfusion of the bone, which might result in bone infarction (Wang et al., 2010). Lastly, it is known that hypercortisolism can lead to endothelial dysfunction (Wagenmakers et al., 2016). Glucocorticoids increase vascular smooth muscle contractility and decrease endothelial-dependent vasodilatation (Walker, 2007). One could hypothesize that this altered vascular tone might lead to decreased blood flow in tissues and thus may contribute to the pathogenesis of glucocorticoid induced bone infarctions.

The golden standard for diagnosing bone infarctions is MRI, while conventional X-ray is less sensitive (Kaste et al., 2011). Bone infarctions can be managed conservatively or surgically. Indication for one or the other depends on stage of disease, size of the lesion, age and comorbidities of the patient (Chan and Mok, 2012). If pain relief is required, core decompression can be performed. During this procedure intramedullary pressure is reduced by making a drill hole, which improves blood flow (Chan and Mok, 2012). To strengthen the bone near the joint, bone impaction grafting can be performed (Rijnen et al., 2006). If the joint has been destroyed by the bone infarction, joint replacement is necessary (Chan and Mok, 2012). Furthermore, bisphosphonates may reduce pain and improve functionality (Luo et al., 2014).

## 4 Conclusion

In conclusion, the patient presented is to our knowledge the first described case of bone infarction of the distal femur in a patient with severe endogenous Cushing's syndrome. In patients with Cushing's syndrome and bone pain, the diagnosis of bone infarction should be considered as it can occur as a rare complication of hypercortisolism.

## Funding sources

This research did not receive any specific grant from funding agencies in the public, commercial, or not-for-profit sectors.

## CRediT authorship contribution statement

Pepijn van Houten: Writing - original draft; Writing – review & editing; Jacky de Rooy: Writing - original draft; Visualization; Ingrid van der Geest: Writing - original draft; Romana Netea-Maier: Writing - review & editing; Annenienke van de Ven: Conceptualization; Supervision; Writing – review & editing.

## Declaration of competing interest

All authors declare no competing interests.
